# The impact of virtual care on drug prescribing practices: A scoping review

**DOI:** 10.1371/journal.pdig.0001192

**Published:** 2026-01-06

**Authors:** Maryann Rogers, Lindsay Hedden, Kimberlyn McGrail, Michael R. Law

**Affiliations:** 1 Centre for Health Services and Policy Research, University of British Columbia, Vancouver, British Columbia, Canada; 2 Faculty of Health Sciences, Simon Fraser University, Burnaby, British Columbia, Canada; 3 School of Medicine, Simon Fraser University, Burnaby, British Columbia, Canada; 4 Department of Community Health Sciences, Cumming School of Medicine, University of Calgary, Calgary, Alberta, Canada; 5 Centre for Health Policy, O’Brien Institute for Public Health, University of Calgary, Calgary, Alberta, Canada; Lahore College for Women University, PAKISTAN

## Abstract

Upon the emergence of COVID-19, virtual alternatives to in-person care developed quickly to meet the need of physicians to maintain medical distancing from patients. Virtual care has since become a mainstay in the landscape of primary care with many physicians providing both virtual and in-person visit options within their practice. However, due to its rapid development, questions have been raised regarding the quality of virtual care compared to its in-person alternative, particularly in terms of prescribing appropriateness. Thus, we examined whether global prescribing patterns differed between virtual and in-person physician visits following the onset of the COVID-19 pandemic. We conducted a scoping review of global literature with narrative synthesis to assess whether and how prescribing patterns differed between virtual and in-person care. This review revealed mixed findings, with the majority of studies reporting no significant difference in medication or antibiotic prescribing rates. Some weak evidence suggested virtual care may be associated with greater adherence to clinical guidelines. However, the predominance of United States based studies and methodological limitations precluded strong conclusions, particularly for the Canadian context. Our scoping review found no consensus in the global literature on how prescribing patterns differ between virtual and in-person care. The methodological weaknesses and limited generalizability of the existing body of evidence highlights the need for further high-quality research in a broader range of settings.

## Authors summary

Virtual care became a prominent form of healthcare delivery following the COVID-19 pandemic. However, such quick implementation may have caused physicians to prescribe differently, and potentially inappropriately, during virtual care visits compared to in-person visits. Here, we investigated whether physician prescribing differs between virtual and in-person visits by conducting a scoping review of the global literature. Our review of global studies found mixed results, with most showing no major differences in prescribing between virtual and in-person care. Some global evidence suggested virtual care may be associated with more appropriate prescriptions. However, reported evidence was largely based out of the United States and many studies were subject to critical risk of bias. These findings indicate that further global research is required to reach consensus on the effects of virtual care on prescribing, particularly outside of the United States with methods less vulnerable to bias.

## Introduction

Virtual care, here defined as a physician visit conducted via telephone, video call, or secure messaging, emerged in the mid to late 20^th^ century. Although Canada played a pioneering role in developing virtual care, the country lagged significantly behind its international peers, particularly the United States, in its adoption until the COVID-19 pandemic in 2020 [1,2]. COVID-19 emerged in China in December 2019 and quickly spread worldwide, prompting the World Health Organization (WHO) to declare it a global pandemic on March 11, 2020 [3]. In response, global policies that directed primary care practitioners to rapidly expand virtual care offerings to maintain “medical distancing” and to implement new guidelines for in-person visits were introduced [4,5].

Virtual care can be split into three delivery modalities: telephone, video call, and secure email/messaging [6]. Telephone visits have been the most common modality in Canada, making up 75% of virtual care visits as of 2022. Virtual and secure email/messaging visits gained traction during the COVID-19 pandemic, with video calls and secure email/messaging making up 21% and 4% of Canadian virtual care visits in 2022, respectively [6].

Virtual care has broad benefits in accessibility for patients living in rural and remote areas, cost-effectiveness for practitioners and insurance schemes, and similar patient satisfaction to in-person primary care [7]. However, the major increase in primary care delivered virtually due to the COVID-19 pandemic was largely implemented as temporary/stop-gap solutions and was not integrated into provider workflows or designed with the patient experience in mind [8]. This rapid implementation may have negatively impacted the ability of health systems to provide adequate training in practicing virtual care for physicians, the consequences of which may persist. At the same time, the rapid uptake of virtual care to high levels may have resulted in practitioners becoming very familiar with it by the end of pandemic restrictions, allowing for similar practice patterns between virtual and in-person modalities.

Since 2020, questions have been raised regarding the quality of care delivered virtually compared to in-person alternatives. Among these are concerns are questions of prescribing appropriateness and adherence to clinical guidelines with virtual visits [9,10]. In general, quality prescribing is rational, evidence based, clear, complete and improves the treatment outcome of the patient [11]. Conversely, low-quality prescribing is defined as prescribing medications that may not produce benefits that outweigh harms, or not prescribing medications that are recommended [12]. Physicians providing virtual care may be affected by limited visual, auditory, and physical assessment capabilities, making more liberal treatment decisions when prescribing medications including unnecessary or prolonged treatments, or conversely, more conservative prescriptions to compensate for the diagnostic uncertainty introduced by virtual care. These concerns are particularly significant for antibiotic prescribing, as inappropriate use of antibiotics—including unindicated treatments—can contribute to the growing issue of antibiotic resistance [12–14].

In response to concerns about the rapid uptake of virtual care and the potential for associated low-quality prescribing, the Canadian Medical Association (CMA) published the *Virtual Care Playbook,* which aimed to help Canadian physicians optimize quality of care in virtual patient encounters [15]. The playbook contains a section on scope of practice, which outlines what medical issues can be safely assessed and treated virtually without compromising the standard of care. This document defines virtual care amenable conditions as those which require only history, gross inspection, and/or data that patients can gather with cameras and common devices, including mental health, skin conditions, and sinus infections [15]. Conditions deemed non-amenable to virtual care include any new and significant emergency symptoms including chest pain and ear pain [15].

The landscape of virtual care changed greatly after the emergence of COVID-19, and it is unclear what the literature has suggested regarding prescribing practices between virtual and in-person care after the pandemic began, as well as whether the implications of this literature apply to a Canadian context where virtual care is less established due to later adoption compared with international peers. Thus, we sought to determine how global trends in medication prescribing and prescription appropriateness, assessed as adherence to clinical prescribing guidelines, differ between virtual and in-person primary care visits. We conducted a scoping review to summarize the research performed in this area and identify current gaps in knowledge. Given the rapid expansion of virtual care from 2020 onward, this review focuses on the time period following the onset of the pandemic, considering the normalization of virtual visits, regulatory changes, and shifts in clinical practice. Understanding these recent trends is critical for informing future policy and ensuring safe, effective prescribing in virtual settings.

## Methods

Our review was guided by the Preferred Reporting Items for Systematic reviews and Meta-Analyses extension for Scoping Reviews (PRISMA-ScR) [16].

### Eligibility criteria

Table 1 details the PICOS (Population, Intervention, Comparison, Outcomes and Study) criteria utilized to determine study eligibility for the review.

**Table 1:** PICOS Eligibility Criteria for Study Inclusion in the Scoping Review.

Criteria	Description
Population	Patients of all ages who had a physician consultation
Intervention	Virtual physician visit (any mode of delivery, including telephone, video, or secure messaging)
Comparator	In-person physician visit
Outcomes	Rate of prescribing Days supply of medication prescribed Type (broad- or narrow-spectrum) of medication prescribed Adherence to clinical guidelines
Study Types	Systematic reviews, randomized controlled trials, non-randomized quantitative studies
Language	English
Date Limits	January 1, 2020 – April 23, 2025

Full-text articles were excluded when they reported only pre-2015 data, examined irrelevant outcomes such as comparisons of virtual prescribing habits between physicians, or used unsuitable designs, including qualitative studies and narrative reviews. Articles conducted in settings outside our scope, such as such as supportive housing or extended care, were also excluded.

### Information sources

To identify potentially relevant documents, we searched Ovid MEDLINE for articles published between January 1, 2020, and April 23, 2025. As our search was in the Canadian context, we chose this time period from 2020 onward as it encompasses the rise of virtual care to prominence in the Canadian healthcare landscape due to the emergence of COVID-19 [17]. The search strategy was initially drafted by an experienced information specialist and subsequently refined by the authors to ensure comprehensiveness and relevance. The search strategy comprised both controlled vocabulary, such as the National Library of Medicine’s MeSH (Medical Subject Headings) terms, and keywords. The main search concepts were virtual care, drug utilization, and prescribing patterns. The finalized search strategy for Ovid MEDLINE is provided in S1 Appendix. In addition to the electronic database search, a backward citation search was conducted on the reference lists of included full-text articles to identify any additional relevant studies.

### Screening

One reviewer (MR) screened citations, reviewed titles and abstracts, and retrieved potentially relevant articles for assessment. The final selection of full-text articles was based on the PICOS criteria outlined in Table 1.

### Extraction

Data were extracted on article characteristics (including country, sample size, and setting), the prescribing outcome(s) of interest addressed (rate of prescribing, days of medication prescribed, type of medication prescribed, adherence to clinical guidelines), and the association of virtual care with the outcome(s). Whether the condition(s) of interest studied in included articles would be deemed amenable to virtual care by the Canadian Medical Association was also classified.

### Critical appraisal

To ensure high- and low- quality studies were not equally weighted in the narrative synthesis, critical appraisal was performed. One author (MR) appraised the quality of the included systematic reviews and observational studies utilizing the AMSTAR2 and ROBINS-I tools, respectively [18,19]. Low-quality elements that recurred across studies were identified and summarized across the included studies.

### Synthesis

Studies were grouped based on the prescribing outcomes they addressed and summarized the direction of effect (increase, no change, decrease, mixed) of virtual care on these outcomes. The synthesized findings were presented narratively, with a graphical representation illustrating the distribution of effect direction within each outcome to provide a clearer visual comparison of the trends observed across studies.

## Results

**Fig 1:**
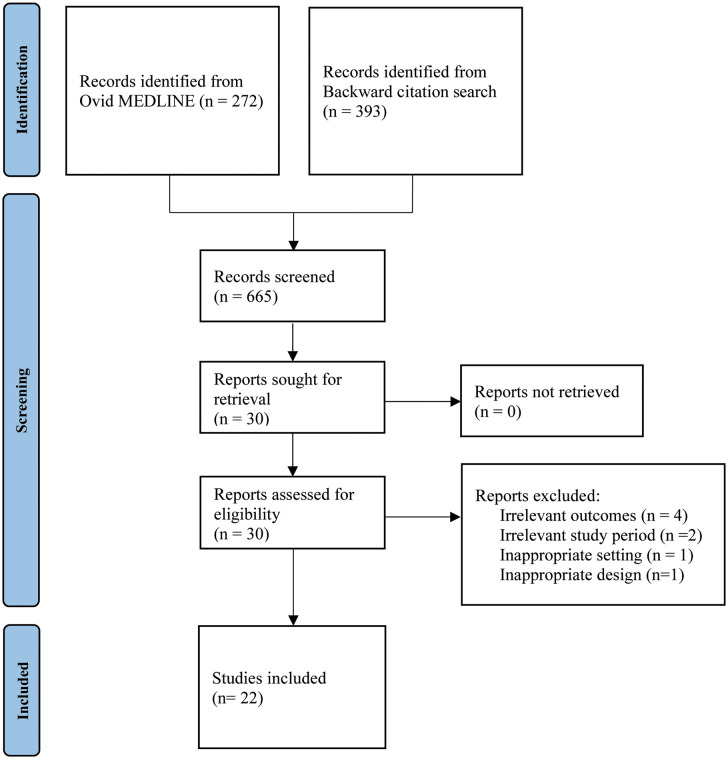
PRISMA flow diagram detailing the record screening process.

Fig 1 provides an overview of the study selection process using a PRISMA flow diagram.

### Study characteristics

Overall, we identified 272 records from the initial search and an additional 393 records from the citations in these original studies. After review, we identified 22 studies: 3 systematic reviews, no randomized controlled trials, and 19 observational studies. All studies focused on an adult population. Minor overlap (n = 5) exists between studies included in the systematic reviews and the observational studies in this scoping review. Characteristics of the included studies are detailed in Table 2 and Table 3, respectively. Due to the somewhat subjective nature of the amenability classification within the CMA’s *Virtual Care Playbook*, particularly for conditions like the common cold where symptoms can fall into both amenable and non-amenable categories, we were unable categorize over half of the conditions studied in our included articles as amenable or non-amenable to virtual care. Thus, we could not make any associations between condition amenability and prescribing outcomes.

**Table 2:** Characteristics of the observational studies included in the review.

Author	Year of Publication	Country	Study Design	Setting	Sample Size	Outcome of Interest
Cueller et al [20]	2022	USA	Retrospective cohort	Primary care	2,400,198 patient visits	Rate of antibiotic prescribing, adherence to clinical guidelines for antibiotic prescribing
Entezarjou et al [21]	2021	Sweden	Retrospective cohort	Primary Care	4,162 patient visits	Rate of antibiotic prescribing, adherence to clinical guidelines for antibiotic prescribing
Fathy et al [22]	2022	USA	Retrospective cohort	Dermatology	9,007 patient visits	Rate of antibiotic prescribing
Gao et al [23]	2025	Australia	Retrospective cross-sectional	Primary care	425,059 patient diagnoses	Rate of antibiotic prescribing
Johnson et al [24]	2021	USA	Retrospective cohort	Primary care	350 patient visits	Rate of antibiotic prescribing, adherence to clinical guidelines for antibiotic prescribing
Khosravi et al [25]	2020	USA	Retrospective chart review	Dermatology	400 patient visits	Rate of antibiotic prescribing
Leong et al [26]	2024	USA	Retrospective cross-sectional	Outpatient cancer rehabilitation physiatry	7,004 patient visits	Rate of general prescribing
Li et al [27]	2021	Wales	Retrospective chart review	Primary care	170 patient visits	Adherence to clinical guidelines for antibiotic prescribing
Martinez et al [28]	2025	USA	Retrospective cross-sectional	Urgent care	88,192 patient visits	Rate of antibiotic prescribing
Miller et al [29]	2021	USA	Cross-sectional	Primary care	5,729 patient visits	Rate of antibiotic prescribing
Mizuno et al [30]	2021	USA	Retrospective cohort	Primary care	20,374 patient visits	Rate of statin prescribing
Murray et al [31]	2020	USA	Retrospective chart review	Primary care	450 patient visits	Rate of antibiotic prescribing
Ostberg et al [32]	2022	USA	Retrospective propensity score matched cohort	Emergency medicine	910 patient visits	Rate of prescribing for patients presenting with chest pain
Patel et al [33]	2021	England	Retrospective cohort	Mental Healthcare	Mean of 37,563 patients registered weekly within a trust’s mental health services over a 52-week period	Rate of antipsychotic and mood stabiliser prescribing
Penza et al [34]	2020	USA	Retrospective chart review	Primary care	505 patient visits	Rate of antibiotic prescribing
Ray et al [35]	2021	USA	Retrospective cohort	Primary care	8,332 patient visits	Rate of antibiotic prescribing, adherence to clinical guidelines for antibiotic prescribing
Wallman et al [36]	2024	Sweden	Retrospective cohort	Primary care	160,238 patient visits	Rate of antibiotic prescribing
Yao et al [37]	2020	USA	Retrospective cohort	Emergency medicine	520 patient visits	Rate of antibiotic prescribing
Yuan et al [38]	2021	USA	Cross-sectional	Cardiology	176,781 patient visits	Rate of prescribing for cardiac conditions

**Table 3:** Characteristics of the systematic reviews included in the review.

Authors	Year of Publication	Country	Setting	Studies Included	Outcome of Interest
Bakhit et al [39]	2021	USA	Primary Care	13	Rate of antibiotic prescribing
Suzuki et al [40]	2021	Multiple	Multiple	23	Rate of antibiotic prescribing, adherence to clinical guidelines for antibiotic prescribing
Turk et al. [41]	2022	USA	Urgent care	7	Rate of antibiotic prescribing, adherence to clinical guidelines for antibiotic prescribing

### Observational studies

The included observational studies were predominantly conducted in US primary care settings. Most focused on the rate of prescribing as the primary outcome, with particular emphasis on antibiotic prescribing rates. The most commonly studied conditions included respiratory tract infection (RTI), addressed in six studies [20,21,23,28,29,35], and urinary tract infection (UTI), addressed in threes studies [20,21,24]. Retrospective cohort and cross-sectional designs were commonly used, with multivariable adjustment serving as the primary method for controlling for covariates. Only one study employed a more robust approach, using propensity score matching to address confounding [32]. While most studies were conducted after the onset of COVID-19, some, particularly those published in 2020, utilized pre-pandemic data. The findings of these studies were generally consistent with broader trends, though overall patterns across studies were quite mixed.

### Observational studies - rate of prescribing

**Fig 2:**
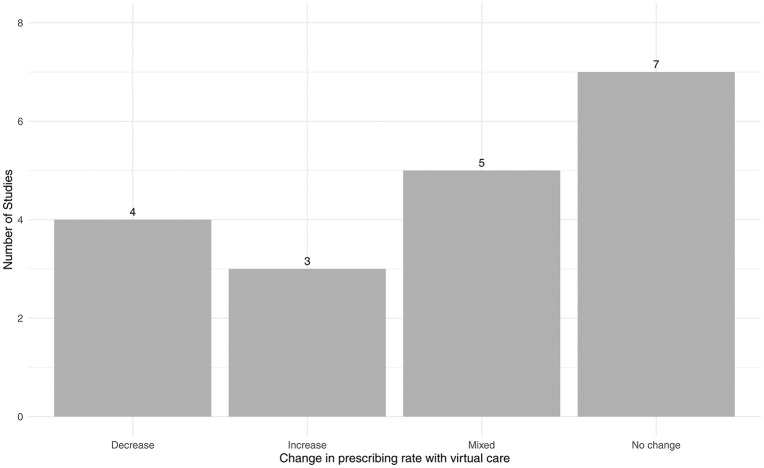
Distribution of the effect of virtual care on rate of prescribing across included observational studies compared to in-person care.

All 19 observational studies reported outcomes on differences in medication prescribing rates between in-person and virtual visits. Fig 2 categorizes the distribution of the effect of virtual care on the prescribing rate in included observational studies. Four studies found that prescribing rates decreased in virtual care visits compared to in-person visits—three in primary care [29,35,36] and one in ambulatory cardiology [38]. In contrast, three studies reported an increase in prescribing rates with virtual care, one in dermatology [25], and one in urgent care, [28] and one in primary care visits for conjunctivitis [34]. The largest proportion of studies (seven) reported no difference in prescribing rates between in-person and virtual care. Among these, three were in primary care [24,27,31], two in emergency medicine [32,37], one in cancer rehabilitation physiatry [26], and one in mental healthcare [33]. Five studies reported mixed results. Cuellar et al. [20] found that virtual care visits were associated with comparable rates of antibiotic use in upper respiratory tract infections, bronchitis, and sinusitis, relative to in-person care, but higher rates in pharyngitis and uncomplicated UTIs. Entezarjou et al. [21] found that antibiotic prescription rates were lower following virtual visits compared with office visits for sore throat and respiratory symptoms, but not for dysuria. Fathy et al. [22] found that among new-patient visits, antibiotics and isotretinoin were more likely to be prescribed in virtual visits compared to in-person visits. However, among returning-patient visits, antibiotics were less likely to be prescribed via virtual care, while isotretinoin remained more likely to be prescribed. Gao et al. [23] saw that antibiotics were less likely to be prescribed in virtual visits compared in in-person visits for sore throat, RTI, and otitis media, but no significant difference was seen between visit modalities for acute bronchitis or acute sinusitis. Mizuno et al. [30] reported that statin prescribing rates were significantly higher during virtual care visits than in-person visits during April 2020 and May 2020. However, there was no significant difference in prescribing rates between virtual and in-person visits during the rest of the 12-month study period.

### Observational studies - rate of antibiotic prescribing

**Fig 3:**
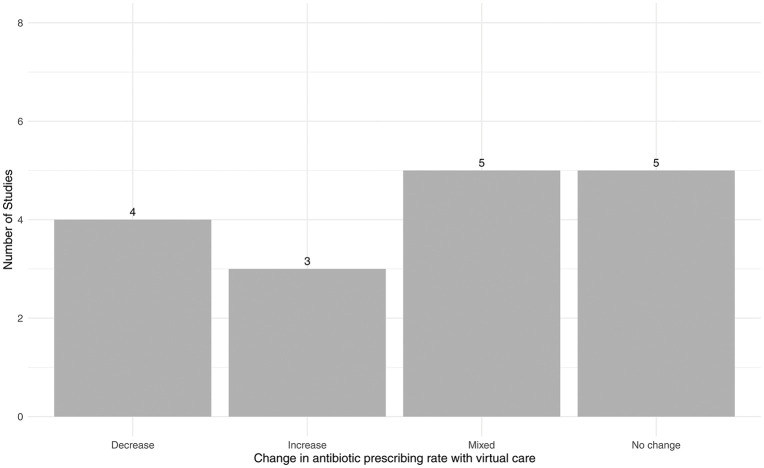
Distribution of the effect of virtual care on rate of antibiotic prescribing across included observational studies compared to in-person care.

17 studies specifically examined antibiotic prescribing rates in virtual versus in-person care. This focus is likely due to concerns about an increase in antibiotic overprescribing, which contributes to antibiotic resistance, during the rapid shift to virtual care in the COVID-19 pandemic [12,13]. Fig 3 displays the distribution of the association of virtual care with the antibiotic prescribing rate. Four studies reported a decrease in antibiotic prescribing with virtual care—three in primary care [29,35,36] and one in ambulatory cardiology [38]. Conversely, three studies reported higher antibiotic prescribing rates in virtual visits with one in dermatology [25] one in urgent care [28], and one in primary care visits for conjunctivitis [34]. The most common finding, reported in five studies, was no significant difference in antibiotic prescribing rates between virtual and in-person care. Among these, two focused on primary care [27,31], two on emergency medicine [32,37], and one on mental healthcare [33]. Five additional studies reported mixed results in antibiotic prescribing patterns between virtual and in-person care, the same as detailed in the previous section [20–23,30].

### Observational studies - days supply and medication type

None of the included studies reported on difference in the days supplied of medication prescribed, or type of medication prescribed between virtual and in-person physician visits.

### Observational studies - adherence to clinical guidelines

Five studies reported on differences in adherence to clinical prescribing guidelines between virtual and in-person care, all of which focused on antibiotic prescribing. Two studies, both conducted in a primary care settings for viral infections and tonsillitis, reported that guideline-concordant antibiotics were more likely to be prescribed in virtual visits compared to in-person visits [20,27]. In contrast, two other studies in a primary care settings for UTI and RTI found no significant difference in adherence to clinical guidelines for antibiotic prescribing between virtual and in-person care [24,35]. Entezarjou et al. reported mixed results, finding that guideline-recommended antibiotics were prescribed between modalities for sore throat visits, however, virtual visits for respiratory symptoms and dysuria were more often prescribed guideline-recommended antibiotics [21].

### Observational studies - critical appraisal

Our critical appraisal of observational studies using the ROBINS-I tool found that the risk of bias related to the classification of interventions, selection of participants, deviations from intended interventions, and outcome measurement was generally low across most included studies. However, bias due to confounding was a serious concern in all studies, primarily because of inadequate selection of confounding variables and the use of analytical methods that did not sufficiently adjust for baseline differences. Additional concerns were identified in several studies that failed to report the extent of missing data or provide an analysis plan to mitigate potential bias in the reported results.

### Systematic reviews

Two of our included three systematic reviews exclusively analyzed US-based studies examining antibiotic prescribing in virtual versus in-person care. Bakhit et al. included 13 studies and Turk et al. included 7 studies [39,41]. The third, Suzuki et al, included a global perspective, however, 21 of its 23 included studies were US-based [40]. All three systematic review focused on common acute conditions, including RTI, UTI, otitis media, and skin infections. The primary studies largely employed unmatched designs and were frequently rated as having a serious risk of bias due to confounding. All reviews primarily included studies conducted prior to the COVID-19 pandemic.

### Systematic reviews - antibiotic prescribing

Two of our included systematic reviews concluded that there was no significant difference in antibiotic prescribing rates between virtual and in-person care [39,41]. The third, Suzuki et al., found that providers prescribed antibiotics more frequently via virtual care for otitis media and pharyngitis but detected no significant difference in the frequencies of antibiotic prescribing between modalities for sinusitis [40]. Generally, heterogeneity in the results of all three reviews limited the ability to draw strong conclusions. No review reported on overall prescribing rates, duration or type of medication prescribed, or adherence to clinical guidelines.

### Systematic reviews - critical appraisal

Our critical appraisal of systematic reviews using the AMSTAR2 tool found that, although the sample of interest was clearly defined using the PICO framework, and the search strategies and reporting of included studies were comprehensive, several critical risk of bias issues were identified. These included the absence of pre-established review methods, lack of justification for the types of studies included, and inadequate assessment of risk of bias, particularly regarding publication bias, in the included studies.

## Discussion

This scoping review identified 22 studies addressing differences in the rate of prescribing and clinical guideline adherence between virtual and in-person physician visits. No included study reported on difference in the days supplied of medication prescribed, or type of medication prescribed between virtual and in-person physician visits, reflecting a notable gap in the current evidence.

Our findings reveal a mixed set of results regarding differences in general medication prescribing, antibiotic prescribing, and adherence to clinical guidelines between virtual and in-person primary care visits. For both general and antibiotic prescribing, the majority of studies found no difference between the two care modalities, suggesting that despite its rapid uptake, virtual care has largely maintained similar rates and appropriateness of prescribing to in-person visits.

This finding was not universal, however. Several studies reported higher prescribing rates in virtual care, indicating a more liberal approach, while others found lower prescribing rates, suggesting more conservative practices. Additionally, some studies showed mixed results, with variations depending on factors such as the type of illness or the timing of the visit, particularly during the height of the pandemic. Further mixed results were seen in how adherence to clinical prescribing guidelines differs between virtual and in-person care, with two studies reporting an increase in guideline concordant prescriptions and an equal number reporting no difference in prescription appropriateness between the modalities. No consistent pattern was observed between study setting and the effect of virtual care.

These mixed results may reflect the serious risk of bias due to confounding in many included studies revealed in our quality appraisal. Such bias could allow unmeasured factors, including patient severity and provider characteristics, to influence the observed patterns, contributing to the inconsistencies reported here. Future research should ensure to utilize study designs, such as rigorous quasi-experimental methods, which adequately control for such confounding.

## Limitations

Our study has several limitations. First, the review was conducted using a single database, Ovid MEDLINE, which may have limited the comprehensiveness of our findings. Relevant articles not indexed in this database may have been missed. In addition, screening was performed by a single reviewer without initial verification or calibration, introducing the potential for selection bias. We also restricted our search to English-language articles published between January 2020 and April 2025, which may have excluded relevant studies outside that time frame or in other languages. Future reviews should expand searches to additional databases and include non-English studies to improve completeness and reduce the risk of selection bias. In addition, our quality assessment found that most included studies were subject to at least one domain at critical risk of bias. In the majority of cases, this risk stemmed from inadequate control for baseline confounding in observational studies due to insufficient covariate selection and basic analytical methods. Many studies relied solely on multivariable adjustment with limited covariates, which may have failed to account for systematic differences between treatment and comparison groups. This raises concerns about bias, the validity of individual study findings, and our ability to draw generalizable conclusions across studies.

In addition, we would have liked to assess whether the conditions examined in the included studies would be considered amenable to virtual care according to CMA guidance so as to not conflate associations between amenable and non-amenable conditions with prescribing patterns. However, this was not possible due to the currently lack of specificity in distinction between amenable and non-amenable conditions in CMA guidelines. The CMA may consider updating these guidelines to provide further detail on its amenability classifications, thereby reducing subjectivity in virtual treatment of conditions which do not fit under a specific category.

Finally, nearly all of the included studies were conducted in the United States, with two additional observational studies each from the United Kingdom and Sweden, as well as one observational study from Australia. While the authors aimed to synthesize a global perspective and generalize the results to Canada and make policy recommendations within a Canadian context, the predominance of studies conducted in the USA, a country with an established landscape of virtual care and a healthcare system that differs significantly from Canada’s and many others worldwide, may limit the generalization of findings beyond the USA.

## Conclusion

The aim of this scoping review was to examine what is globally known about differences in prescribing practices, specifically the rate of prescribing, days prescribed, type of medication prescribed, and adherence to clinical guidelines, between virtual and in-person physician visits. Overall, our findings indicate that prescribing practices in virtual care following the emergence of COVID-19 largely mirror those seen in in-person care. However, the highly mixed results concentrated in the USA suggest the need for additional research to reach a consensus on how prescribing practices differ between virtual and in-person care. In addition, we found no evidence regarding the duration or type of medication prescribed. The impact of virtual care on prescribing rates and adherence to clinical guidelines showed mixed effects, with the majority of studies reporting no significant difference in general medication or antibiotic prescribing rate. Some evidence suggested virtual care may be associated with greater adherence to clinical guidelines.

There is insufficient evidence to draw strong conclusions or make policy recommendations, particularly within the Canadian context, as nearly all included studies were conducted in the USA. Additionally, methodological concerns limit the validity of many of the included observational studies, further precluding our ability to draw conclusions. Further high-quality research with analytic methods better suited to control for baseline confounding is needed to understand the impact of virtual care on prescribing rates and adherence to clinical guidelines, as well as to explore how virtual care affects the duration and type of medications prescribed.

## Supporting information

S1 Appendix Ovid MEDLINE Search Strategy. (DOCX)

S1 Checklist PRISMA Checklist - Reproduced from: Tricco AC, Lillie E, Zarin W, O’Brien KK, Colquhoun H, Levac D, et al. *PRISMA Extension for Scoping Reviews (PRISMA-ScR): Checklist and Explanation.* *Ann Intern Med*. 2018;169:467–473. Licensed under CC BY 4.0.
(PDF)

## References

[1] Virtual Care Task Force. Virtual Care - Recommendations for Scaling Up Virtual Medical Services [Internet]. 2020 Feb. Available from: https://www.cfpc.ca/CFPC/media/Images/PDF/VCTF-report-Final-ENG-Feb-11-20.pdf

[2] Canadian Institute for Health Information. How Canada Compares: Results From the Commonwealth Fund’s 2020 International Health Policy Survey of the General Population in 11 Countries [Internet]. Ottawa, On: CIHI; 2021. Available from: https://www.cihi.ca/sites/default/files/document/how-canada-compares-cmwf-survey-2020-chartbook-en.pdf

[3] Coronavirus disease (COVID-19) pandemic [Internet]. [cited 2025 Mar 18]. Available from: https://www.who.int/europe/emergencies/situations/covid-19

[4] Rawaf S, Allen LN, Stigler FL, Kringos D, Quezada Yamamoto H, van Weel C, et al. Lessons on the COVID-19 pandemic, for and by primary care professionals worldwide. Eur J Gen Pract. 2020;26(1):129–33. doi: 10.1080/13814788.2020.1820479 32985278 PMC7534357

[5] Matenge S, Sturgiss E, Desborough J, Hall Dykgraaf S, Dut G, Kidd M. Ensuring the continuation of routine primary care during the COVID-19 pandemic: a review of the international literature. Fam Pract. 2022;39(4):747–61. 34611708 10.1093/fampra/cmab115 PMC8515263

[6] Canada Health Infoway. Canadians’ Health Care Experiences During COVID-19: Uptake of Virtual Care. 2022 Apr 22; Available from: https://www.infoway-inforoute.ca/en/component/edocman/3828-canadians-health-care-experiences-during-covid-19/view-document?Itemid=103

[7] Li C (Zhirui), Borycki EM, Kushniruk AW. Connecting the world of healthcare virtually: a scoping review on virtual care delivery. Healthcare. 2021;9(10):1325. 34683005 10.3390/healthcare9101325 PMC8544348

[8] Canada H. Virtual care policy framework [Internet]. 2022 [cited 2024 Sept 3]. Available from: https://www.canada.ca/en/health-canada/corporate/transparency/health-agreements/bilateral-agreement-pan-canadian-virtual-care-priorities-covid-19/policy-framework.html

[9] Hui D, Dolcine B, Loshak H. Approaches to Evaluations of Virtual Care in Primary Care [Internet]. Canada’s Drug Agency; 2022 Jan [cited 2025 Feb 16]. Available from: https://www.cda-amc.ca/evaluations-virtual-care 37797106

[10] Hardcastle L, Ogbogu U. Virtual care: enhancing access or harming care?. Healthc Manage Forum. 2020;33(6):288–92. 32686506 10.1177/0840470420938818 PMC7372098

[11] Suthar JV, Patel VJ. Assessment of quality of prescribing in patients of hypertension at primary and secondary health care facilities using the Prescription Quality Index (PQI) tool. Indian J Pharmacol. 2014;46(5):480–4. doi: 10.4103/0253-7613.140576 25298574 PMC4175881

[12] Mekonnen AB, Redley B, de Courten B, Manias E. Potentially inappropriate prescribing and its associations with health-related and system-related outcomes in hospitalised older adults: A systematic review and meta-analysis. Br J Clin Pharmacol. 2021;87(11):4150–72. doi: 10.1111/bcp.14870 34008195 PMC8597090

[13] Pfister T, Schröder S, Heck J, Bleich S, Krüger THC, Wedegärtner F, et al. Potentially inappropriate prescriptions of antibiotics in geriatric psychiatry—a retrospective cohort study. Front Psychiatry [Internet]. 2024 [cited 2024 Mar 3];14. Available from: https://www.frontiersin.org/journals/psychiatry/articles/10.3389/fpsyt.2023.1272695 10.3389/fpsyt.2023.1272695 PMC10803574 38264634

[14] Chokshi A, Sifri Z, Cennimo D, Horng H. Global Contributors to Antibiotic Resistance. J Glob Infect Dis. 2019;11(1):36–42. doi: 10.4103/jgid.jgid_110_18 30814834 PMC6380099

[15] Canadian Medical Association, College of Family Physicians of Canada, Royal College of Physicians and Surgeons of Canada. Virtual Care Playbook [Internet]. Ottawa: The Association; 2021. Available from: https://digitallibrary.cma.ca/link/digitallibrary52

[16] Tricco AC, Lillie E, Zarin W, O’Brien KK, Colquhoun H, Levac D. PRISMA Extension for Scoping Reviews (PRISMA-ScR): Checklist and Explanation. Ann Intern Med. 2018;169(7):467–73. 30178033 10.7326/M18-0850

[17] Glazier RH, Green ME, Wu FC, Frymire E, Kopp A, Kiran T. Shifts in office and virtual primary care during the early COVID-19 pandemic in Ontario, Canada. Can Med Assoc J. 2021;193(6):E200. 10.1503/cmaj.202303 PMC7954541 33558406

[18] Shea BJ, Reeves BC, Wells G, Thuku M, Hamel C, Moran J, et al. AMSTAR 2: a critical appraisal tool for systematic reviews that include randomised or non-randomised studies of healthcare interventions, or both. BMJ. 2017;358:j4008. 10.1136/bmj.j4008 PMC5833365 28935701

[19] Sterne JA, Hernán MA, Reeves BC, Savović J, Berkman ND, Viswanathan M. ROBINS-I: a tool for assessing risk of bias in non-randomised studies of interventions. BMJ. 2016;355:i4919. doi: 10.1136/bmj.i4919 PMC5062054 27733354

[20] Cuellar A, Pomeroy JML, Burla S, Jena AB. Quality of antibiotic prescribing in a large direct-to-patient telehealth program: an observational study. J Gen Intern Med. 2022;37(12):3202–4. 35048296 10.1007/s11606-021-07354-8 PMC8769091

[21] Entezarjou A, Calling S, Bhattacharyya T, Nymberg VM, Vigren L, Labaf A, et al. Antibiotic Prescription Rates After eVisits Versus Office Visits in Primary Care: Observational Study. JMIR Medical Informatics. 2021;9(3):e25473. doi: 10.2196/25473 PMC8077790 33720032

[22] Fathy R, Briker S, Rodriguez O, Barbieri JS. Comparing antibiotic prescription rates between in-person and telemedicine visits. J Am Acad Dermatol. 2022;87(2):438–40. 34499992 10.1016/j.jaad.2021.08.064 PMC9222146

[23] Gao Y, Magin P, Tapley A, Holliday E, Dizon J, Fisher K, et al. Prevalence of Antibiotic Prescribing for Acute Respiratory Tract Infection in Telehealth Versus Face-to-Face Consultations: Cross-Sectional Analysis of General Practice Registrars’ Clinical Practice. J Med Internet Res. 2025;27:e60831. doi: 10.2196/60831 40080812 PMC11950701

[24] Johnson KL, Dumkow LE, Salvati LA, Johnson KM, Yee MA, Egwuatu NE. Comparison of diagnosis and prescribing practices between virtual visits and office visits for adults diagnosed with uncomplicated urinary tract infections within a primary care network. Infect Control Hosp Epidemiol. 2021;42(5):586–91. doi: 10.1017/ice.2020.1255 33118916

[25] Khosravi H, Zhang S, Siripong N, Moorhead A, English III JC. Comparing acne follow-up: teledermatology versus outpatient dermatology visits. Dermatol Online J. 2020;26(4). 32621674

[26] Leong D, Ng A, Chang P, Zheng J, Wilson R, Chen ME, et al. Telemedicine impact on patient disparities and physician practice patterns in cancer rehabilitation: A multicenter retrospective study. PM R. 2024;16(12):1298–306. doi: 10.1002/pmrj.13199 38864328 PMC11626559

[27] Li C, Ong C, Morris A, Woollons I, Ashfaq A, Jagatia R. Evaluating the Appropriateness of Antibiotic Treatment of Tonsillitis during COVID-19 in the North Wale Primary Healthcare Setting. J Prim Care Community Health. 2021;12. doi: 10.1177/21501327211003687 PMC7983464 33733905

[28] Martinez KA, Deshpande A, Stanley E, Rothberg MB. Antibiotic prescribing for respiratory tract infections in urgent care: a comparison of in-person and virtual settings. Clin Infect Dis. 2025;80(1):7–13. 39078065 10.1093/cid/ciae396

[29] Miller LE, Bhattacharyya N. Antibiotic Prescribing for Acute Rhinosinusitis: In-Person Versus Virtual Visits During Covid-19. Laryngoscope. 2021;131(7):E2121–4. doi: 10.1002/lary.29323 33296088

[30] Mizuno A, Patel MS, Park SH, Hare AJ, Harrington TO, Adusumalli S. Statin prescribing patterns during in-person and telemedicine visits before and during the COVID-19 pandemic. Circ Cardiovasc Qual Outcomes. 2021;14(10):e008266. 10.1161/CIRCOUTCOMES.121.008266 PMC8530894 34551588

[31] Murray MA, Penza KS, Myers JF, Furst JW, Pecina JL. Comparison of eVisit Management of Urinary Symptoms and Urinary Tract Infections with Standard Care. Telemed J E Health. 2020;26(5):639–44. doi: 10.1089/tmj.2019.0044 31313978

[32] Ostberg N, Ip W, Brown I, Li R. Impact of telemedicine on clinical practice patterns for patients with chest pain in the emergency department. Int J Med Inf. 2022;161:104726. doi: 10.1016/j.ijmi.2022.104726 PMC8864961 35228006

[33] Patel R, Irving J, Brinn A, Broadbent M, Shetty H, Pritchard M, et al. Impact of the COVID-19 pandemic on remote mental healthcare and prescribing in psychiatry: an electronic health record study. BMJ Open. 2021;11(3):e046365. doi: 10.1136/bmjopen-2020-046365 33785494 PMC8728386

[34] Penza KS, Murray MA, Myers JF, Maxson J, Furst JW, Pecina JL. Treating pediatric conjunctivitis without an exam: An evaluation of outcomes and antibiotic usage. J Telemed Telecare. 2020;26(1–2):73–8. 30153768 10.1177/1357633X18793031

[35] Ray KN, Martin JM, Wolfson D, Schweiberger K, Schoemer P, Cepullio C, et al. Antibiotic Prescribing for Acute Respiratory Tract Infections During Telemedicine Visits Within a Pediatric Primary Care Network. Acad Pediatr. 2021;21(7):1239–43. doi: 10.1016/j.acap.2021.03.008 33741531

[36] Wallman A, Svärdsudd K, Bobits K, Wallman T. Antibiotic Prescribing by Digital Health Care Providers as Compared to Traditional Primary Health Care Providers: Cohort Study Using Register Data. J Med Internet Res. 2024;26:e55228. doi: 10.2196/55228 38924783 PMC11237768

[37] Yao P, Clark S, Gogia K, Hafeez B, Hsu H, Greenwald P. Antibiotic Prescribing Practices: Is There a Difference Between Patients Seen by Telemedicine Versus Those Seen In-Person?. Telemed J E Health. 2020;26(1):107–9. doi: 10.1089/tmj.2018.0250 30762493

[38] Yuan N, Pevnick JM, Botting PG, Elad Y, Miller SJ, Cheng S. Patient use and clinical practice patterns of remote cardiology clinic visits in the era of COVID-19. JAMA Netw Open. 2021;4(4):e214157. doi: 10.1001/jamanetworkopen.2021.4157 PMC8022216 33818619

[39] Bakhit M, Baillie E, Krzyzaniak N, van Driel M, Clark J, Glasziou P, et al. Antibiotic prescribing for acute infections in synchronous telehealth consultations: a systematic review and meta-analysis. BJGP Open. 2021;5(6):BJGPO.2021.0106. doi: 10.3399/BJGPO.2021.0106 34497096 PMC9447298

[40] Suzuki H, Marra AR, Hasegawa S, Livorsi DJ, Goto M, Perencevich EN, et al. Outpatient antibiotic prescribing for common infections via telemedicine versus face-to-face visits: Systematic literature review and meta-analysis. Antimicrob Steward Healthc Epidemiol. 2021;1(1):e24. doi: 10.1017/ash.2021.179 36168456 PMC9495625

[41] Turk K, Jacobson Vann J, Oppewal S. Antibiotic prescribing patterns and guideline-concordant management of acute respiratory tract infections in virtual urgent care settings. J Am Assoc Nurse Pract. 2022;34(6):813–24. doi: 10.1097/JXX.0000000000000705 35472013

