# Supplementary material for: The impact of virtual care on drug prescribing practices: A scoping review
Source: PLOS Digit Health. 2026 Jan 6;5(1):e0001192. doi: 10.1371/journal.pdig.0001192 (PMC12773812; doi:10.1371/journal.pdig.0001192)
Supplement: S1 Appendix — (DOCX) [file pdig.0001192.s001.docx]

# S1 Appendix – Ovid MEDLINE Search Strategy

| **#** | **Query** | **Results from 23 Apr 2025** |
| --- | --- | --- |
| 1 | Telemedicine/ | 44,040 |
| 2 | (Virtual Care or Telemedicine or Virtual Visit* or E-Health or Online Care or Online Visit* or Ehealth).mp. [mp=title, book title, abstract, original title, name of substance word, subject heading word, floating sub-heading word, keyword heading word, organism supplementary concept word, protocol supplementary concept word, rare disease supplementary concept word, unique identifier, synonyms, population supplementary concept word, anatomy supplementary concept word] | 67,689 |
| 3 | 1 or 2 | 67,689 |
| 4 | practice patterns, nurses'/ or practice patterns, physicians'/ | 72,767 |
| 5 | Drug Utilization/ | 21,711 |
| 6 | (Prescribing pattern* or Prescribing practice*).mp. [mp=title, book title, abstract, original title, name of substance word, subject heading word, floating sub-heading word, keyword heading word, organism supplementary concept word, protocol supplementary concept word, rare disease supplementary concept word, unique identifier, synonyms, population supplementary concept word, anatomy supplementary concept word] | 9,793 |
| 7 | 4 or 5 or 6 | 96,185 |
| 8 | 3 and 7 | 484 |
| 9 | limit 8 to (english language and year="2020 -Current") | 272 |
